# The Koolungar ( *Children* ) Moorditj ( *Strong* ) Healthy Skin Project Part I: Conducting First Nations Research in Pediatric Dermatology

**DOI:** 10.1111/pde.70018

**Published:** 2025-09-18

**Authors:** Bernadette M. Ricciardo, Jacinta Walton, Noel Nannup, Dale Tilbrook, Heather‐Lynn Kessaris, Ainslie Poore, Taleah Ugle, Carol Michie, Brad Farrant, Cheryl Bridge, Kelli McIntosh, S. Prasad Kumarasinghe, Asha C. Bowen

**Affiliations:** ^1^ University of Western Australia Crawley Australia; ^2^ The Kids Research Institute Australia Nedlands Australia; ^3^ Perth Children's Hospital Nedlands Australia; ^4^ Fiona Stanley Hospital Murdoch Australia

**Keywords:** child, dermatology, first nations, indigenous peoples, research ethics, skin Australian aboriginal and Torres Strait islander peoples

## Abstract

Integrating First Nations knowledge systems and Western research methodologies recognizes the strength, experience, and insight of First Nations peoples in addressing health issues in their communities. In research, this includes projects being led by First Nations Elders and peoples, including First Nations researchers in the team, and collecting data in ways that reflect First Nations ways of knowing, being, and doing. In this paper, we reflect upon the Koolungar (*children*) Moorditj (*strong*) Healthy Skin Project; operational in Perth and Bunbury, Western Australia, Australia, where the traditional custodians are the Noongar Aboriginal people. This Aboriginal Elder co‐designed project is presented as a case study to illustrate the practical use of The Kids Research Institute Australia Standards for the Conduct of Aboriginal Health Research, in striving towards best practice in Aboriginal pediatric dermatology research. It leads into The Koolungar (*children*) Moorditj (*strong*) Healthy Skin Project Part II manuscript, in which we present cross‐sectional studies of Aboriginal children attending community skin screening weeks.

## 1 Introduction

Culture is central to Australian Aboriginal and Torres Strait Islander (hereafter, Aboriginal) identities. Encompassing the complexities and intersubjectivity of Aboriginal culture, language, and history is essential in health research. This is achieved by shifting the research paradigm from research *on or about* Aboriginal peoples to research *with and by* Aboriginal peoples [1]. This involves research being led by Aboriginal people, having Aboriginal researchers in the team, and collecting data in ways that reflect Aboriginal ways of knowing, being, and doing [2].

The Kulunga (*children*) Aboriginal Team at The Kids Research Institute Australia (The Kids, formerly known as Telethon Kids Institute) has led the design, development, and implementation of the Standards for the Conduct of Aboriginal Health Research (hereinafter, The Aboriginal Standards) [3]. Through community voices, The Aboriginal Standards ensure research of importance to Aboriginal peoples' health is conducted with and by Aboriginal peoples. First Nations people in Australia have been systematically disadvantaged by the processes and policies implemented since colonization in 1788. This historical legacy has favored dominant Western knowledge systems over traditional First Nations knowledge. In deconstructing the academy, we seek to embrace two‐way learning and value both knowledge systems equally. The Aboriginal Standards guide researchers to adopt a community and cultural lens to the co‐design, implementation, and evaluation of projects; improving community engagement and participation [4, 5, 6, 7].

The Healthy Skin and Acute Rheumatic Fever Prevention research team at The Kids worked with Aboriginal Elders, community members, and Aboriginal Community Controlled Health Organizations (ACCHO) on the Koolungar (*children*) Moorditj (*strong*) Healthy Skin (KMHS) Project to describe skin health in urban‐living Aboriginal children in Western Australia (WA) (Box 1) (*see The Koolungar (children) Moorditj (strong) Healthy Skin Project Part II*) [8, 9, 10, 11, 13]. Here, we present the KMHS Project as a novel case study to illustrate the practical use of The Aboriginal Standards in striving towards best practice in Aboriginal pediatric dermatology research.

BOX 1 **Summary of the Koolungar Moorditj Healthy Skin (KMHS) Project.**

The Koolungar (*children*) Moorditj (*strong*) Healthy Skin Project is the first Australian co‐designed research‐service study to comprehensively describe skin health in urban‐living Aboriginal children and young people (CYP). Guided by principles of respect, reciprocity, capacity building, and community involvement, and in collaboration with Aboriginal Elder researchers, Aboriginal community advisory groups, and Aboriginal Community Controlled Health Organizations, we conducted observational studies to examine skin health at community (Part II) [8], primary care [9] and specialist dermatology levels [10]. The results identified the skin health priorities for urban‐living Aboriginal CYP, leading to culturally relevant educational and health promotion resources (Supporting Information Appendix 3) [11, 18], treatment guidelines [19], successful funding applications for sustainable dermatology services, and future community‐identified research priorities.

## 2 Aboriginal Research Standards (Figures 1 and 2)

**FIGURE 1:**
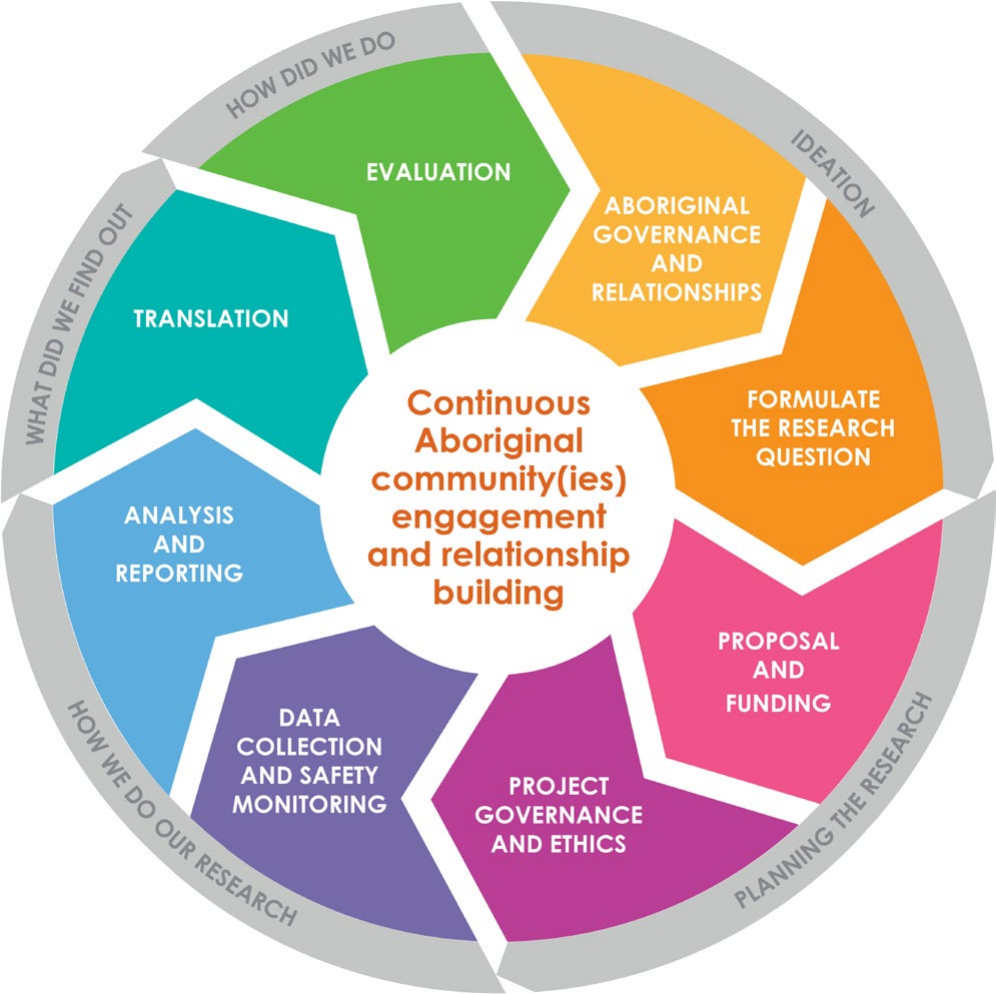
Aboriginal Research Standards process at The Kids Research Institute Australia. Figure used with permission from the Kulunga Aboriginal Team at The Kids.

**FIGURE 2:**
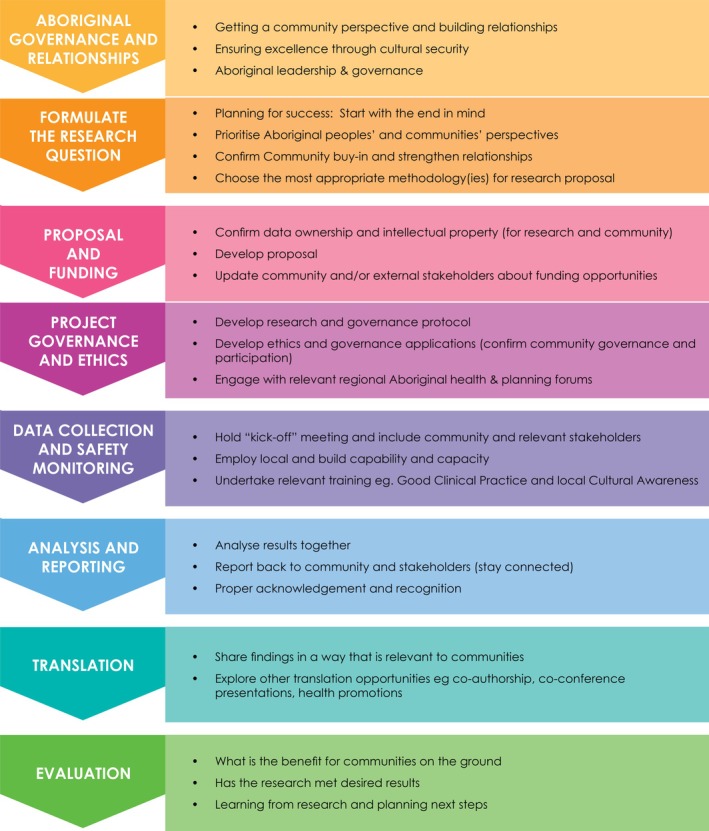
Aboriginal Research Standards and subsequent Actions. Figure reproduced with permission from the Kulunga Aboriginal Team at The Kids.

### 2.1 Standard 1: Aboriginal Governance and Relationships

The Aboriginal Standards guide us to prioritize Aboriginal leadership and governance in the research team in the ideation phase: setting the project up for excellence through cultural security. Community engagement with Aboriginal Elders began in 2019 to determine the interest, scope, and importance of skin health for urban‐living Aboriginal children. Following several meetings, two Noongar Elders (*Traditional Custodians of the south‐west corner of WA*) joined the research team. They provided cultural leadership and governance in project design and execution to align with Aboriginal values and provided oversight of all project outputs for cultural accuracy.

**FIGURE 3:**
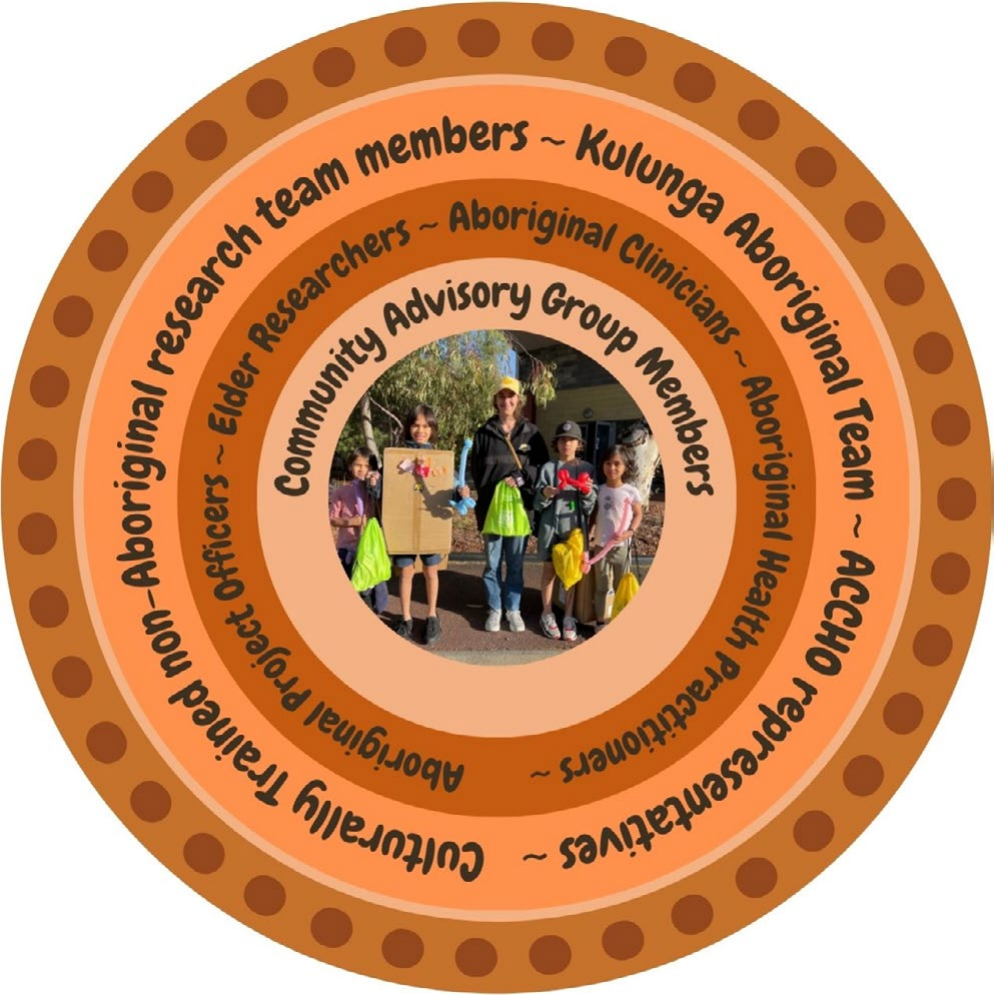
The structure of Aboriginal leadership and governance in the Koolungar Moorditj Healthy Skin (KMHS) Project, with Aboriginal children and communities central to all decision making.

Funding was prioritized for cultural awareness training and community involvement, leading to the appointment of nine Aboriginal community advisory group (CAG) members representing the two study sites of Whadjuk (*Perth*) and Wardandi (*Bunbury*) Noongar Boodjar (*land/place*). They strengthened the project's Aboriginal governance, providing local leadership and cultural guidance on specific community considerations. Funding also enabled the expansion of the Aboriginal research and clinical team, including a full‐time Aboriginal project officer and four Aboriginal health practitioners (AHP). Cultural governance at The Kids was supported by The Kulunga Aboriginal Team, including cultural awareness training and community engagement (Figure 3).

### 2.2 Standard 2: Formulate the Research Question

The Aboriginal Standards provide guidance on prioritizing Aboriginal peoples' perspectives in formulating research questions and methodologies. Over several meetings with Elder researchers, the connection between healthy skin and a healthy environment emerged as a priority for investigation, and culturally sensitive questions to address this were built into the protocol.

Elder researchers considered a research‐service model essential to the study design to ensure study participants received timely specialist assessment and treatment when skin conditions were identified. This approach reflects the philosophy of the late Australian ophthalmology professor, Fred Hollows, who believed there should be “no research without service” highlighting the ethical obligation to provide direct health benefits alongside data collection [14]. Embedding care within research promotes cultural safety, builds trust, and addresses health inequities [15]. In response, partnerships were formed with two urban ACCHOs to establish pediatric dermatology clinics at each study site, coordinated by ACCHO‐embedded AHPs, to enhance culturally safe care.

### 2.3 Standard 3: Proposal and Funding

Recognition of data ownership is critical in research proposals and funding applications, as outlined in The Aboriginal Standards. The KMHS Project sought to align with Indigenous data sovereignty principles, which assert Indigenous rights to govern data's creation, collection, ownership, and application [16]. Consistent with this, a strong Aboriginal workforce was involved at every stage of the project. In protocol development, careful consideration was given to what data were collected and how. Participant information and consent clearly outlined the type of data collected and how it would be used, allowing caregivers to opt in or out of various study components.

Following best practice, data were made available to participating ACCHOs to support funding applications for ongoing service delivery. Dissemination of results was guided by Elder researchers, CAG members, and ACCHO representatives to ensure shared information was contextualized and respectful. Open access was prioritized for all peer‐reviewed publications, ensuring data remained accessible to relevant communities [8, 9, 10, 11, 13].

### 2.4 Standard 4: Project Governance and Ethics

The Aboriginal Standards guide us to develop research protocols through co‐design and engagement with relevant Aboriginal ethics and governance oversight committees. Research co‐design refers to the meaningful involvement of research users during study planning [12]. It represents best practice for research with First Nations peoples and communities, recognizing the value of First Nations knowledge systems and nurturing two‐way learning and collaboration. As named investigators, Elder researchers were integral to the creation of all study elements, including the project title and guiding principles: *respect, reciprocity, capacity building*, *and community involvement*. Following extensive consultation and co‐design, support was obtained from partnering ACCHOs, and the project received ethics approval from the WA Aboriginal Health Ethics Committee.

### 2.5 Standard 5: Data Collection and Safety Monitoring

Employment and capacity building of Aboriginal peoples to undertake data collection are prioritized in The Aboriginal Standards. In the KMHS Project, funding supported employment and capacity building of Aboriginal community, research, and clinical team members. Their involvement in all participant‐facing activities (including questionnaires, skin examination and healthy promotion) provided cultural safety.

Place‐based cultural awareness training with Whadjuk and Wardandi leaders was included in the budget, upskilling non‐Aboriginal team members to better understand the diversity of Aboriginal peoples and cultures. Prior to community skin screening weeks, briefing sessions were held to address cultural awareness and safety considerations [8]. Daily debrief sessions led by the Aboriginal project officer during the screening weeks further strengthened cultural safety.

The KMHS Project utilized a yarning style of communication in all data collection activities with families, adopting a culturally grounded, conversational approach centered around Aboriginal oral traditions [17]. Yarning supports trust‐building and relational engagement before transitioning into more focused, topic‐specific discussions. It enhances cultural safety by privileging Aboriginal voices and ensuring participants feel respected, heard, and understood.

### 2.6 Standard 6: Analysis and Reporting

The Aboriginal Research Standards guide us to analyze results together, report back to community and stakeholders, and ensure proper acknowledgement. The KMHS team developed a collaborative way to analyze results, involving a group reading of all drafted manuscripts. This enabled open discussion of the results and culturally informed interpretation. The perspectives of Elder researchers, ACCHO representatives, and Aboriginal team members were reflected, presenting a strengths‐based perspective. In publications, all Aboriginal peoples and organizations involved were formally recognized for their contributions [8, 9, 10, 11, 13].

### 2.7 Standard 7: Translation

The KMHS Project employed different translational approaches to share findings in ways relevant to the community, per The Aboriginal Standards. The research team, community members, and supporters were kept informed via newsletters (Supporting Information Appendices 1 and 2). Research findings were presented to ACCHO boards, and thank you letters with key results were shared with participating families [8]. The findings were co‐presented at Aboriginal community forums (*n* = 8), and local (*n* = 12), national and international (*n* = 14) conferences.

**FIGURE 4:**
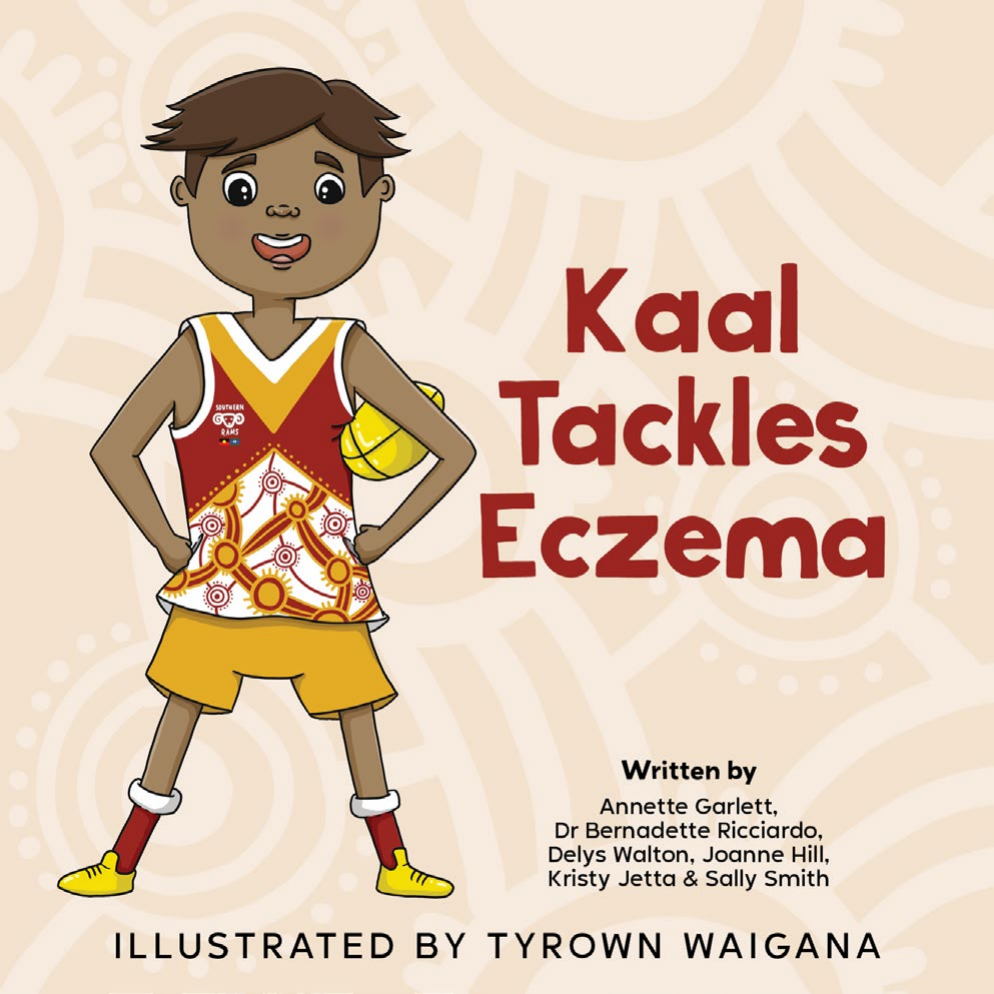
Front cover of *Kaal Tackles Eczema*.

The CAGs led the development of several health promotion resources on *moorditj* skin, used as culturally relevant tools to share results (Supporting Information Appendix 3) [11]. With KMHS Project results revealing eczema affecting nearly 20% of urban‐living Aboriginal children and close to half of these reporting severe symptoms impacting sleep, CAG members prioritized the development of the first‐ever eczema storybook relevant to Aboriginal children (Figure 4). A strengths‐based story, *Kaal Tackles Eczema*, observes the child protagonist demonstrating resilience while learning to cope with eczema [18]. Children and families are empowered with knowledge of eczema to alleviate eczema's impact.

Over the course of many meetings, all elements of *Kaal Tackles Eczema* were meticulously workshopped with the CAG members. Every detail—from character names, interests, occupations, clothing, hair, and skin tone—was carefully considered. All named family members have Noongar names, including “Kaal” meaning fire. The appearance of Kaal's eczema reflects that typically seen in a child of his skin color; likewise, the packaging of skin care products and medicines represents those commonly available in Australia. The CAG also wanted to incorporate public health messaging in the illustrations—including sun protection, physical activity, age‐appropriate child car seats, and the absence of electronic devices. Evaluation of *Kaal Tackles Eczema* with Aboriginal families of children with eczema is now underway (https://infectiousdiseases.thekids.org.au/our‐research/vaccine‐trials‐group/current‐studies/moorditj‐marp/). The findings will inform the development of further healthy skin story books, focusing on other community‐prioritized skin concerns.

### 2.8 Standard 8: Evaluation

As per The Aboriginal Standards, the KMHS Project evaluated outcomes and promoted reflective practice. The research‐service model delivered beneficial service provision with timely dermatological treatment. Accessible and culturally relevant clinical factsheets improved skin health knowledge, strengthening the community's capacity to manage skin disease [11]. The involvement of AHPs in the clinics provided on‐the‐job dermatology training, of ongoing benefit to the communities these ACCHOs serve. The results helped secure funding for sustainable ACCHO‐embedded dermatology clinics and informed the second edition of the *National Healthy Skin Guideline (NHSG)* (thekids.org.au) [19]. Emerging from the KMHS Project, several new skin health projects are underway, prioritizing Aboriginal voices in seeking solutions to prevent, identify, and treat skin disease.

## 3 Conclusion

Integrating First Nations knowledge with Western research methodologies enhances the health and wellbeing of First Nations peoples by valuing their strengths, experiences, and insights. It is a mechanism for empowerment and self‐determination. Co‐design with Aboriginal Elders and community members on the KMHS Project has been vital in ensuring culturally safe research. Guided by The Aboriginal Standards, the KMHS Project has resulted in improved dermatology services, treatment guidelines, and educational resources for Aboriginal children, their families, and communities.

## Consent

The authors have nothing to report.

## Conflicts of Interest

The authors declare no conflicts of interest.

## Supporting information


**Appendix 1.** KMHS Project Newsletter—1st edition, April 2023
**Appendix 2**. KMHS Project Newsletter—2nd edition, April 2024
**Appendix 3**. KMHS Project Resources—community led and co‐created.

## Data Availability

The data that support the findings of this study are available on request from the corresponding author. The data are not publicly available due to privacy or ethical restrictions.
