# Supplementary material for: The Koolungar ( Children ) Moorditj ( Strong ) Healthy Skin Project Part I: Conducting First Nations Research in Pediatric Dermatology
Source: Pediatr Dermatol. 2025 Sep 18;43(1):20–5. doi: 10.1111/pde.70018 (PMC12854924; doi:10.1111/pde.70018)
Supplement: Supplementary file 1 — Appendix 1. KMHS Project Newsletter—1st edition, April 2023 Appendix 2. KMHS Project Newsletter—2nd edition, April 2024 Appendix 3. KMHS Project Resources—community led and co‐created. [file PDE-43-20-s001.docx]

Table of Contents

[1	Appendix 1: KMHS Project Newsletter – 1^st^ Ed., April 2023	2](#_Toc202198854)

[2	Appendix 2: KMHS Project Newsletter – 2^nd^ Ed., April 2024	12](#_Toc202198855)

[3	Appendix 3: KMHS Project Resources – community led and co-created	29](#_Toc202198856)

# Appendix 1: KMHS Project Newsletter – 1^st^ Ed., April 2023

# Appendix 2: KMHS Project Newsletter – 2^nd^ Ed., April 2024

# Appendix 3: KMHS Project Resources – community led and co-created
